# Characterizing Stair Ambulation Kinetics and the Effects of Dual Tasking in Parkinson’s Disease

**DOI:** 10.3390/jcm14165830

**Published:** 2025-08-18

**Authors:** Sumner V. Jones, Colin Waltz, Eric Zimmerman, Mandy Miller Koop, Karissa Hastilow, Jay L. Alberts

**Affiliations:** 1 Department of Biomedical Engineering, Lerner Research Institute, Cleveland Clinic, 9500 Euclid Ave., Cleveland, OH 44195, USA; joness133@ccf.org (S.V.J.); waltzc@ccf.org (C.W.); koopm@ccf.org (M.M.K.); 2 Center for Neurological Restoration, Neurological Institute, Cleveland Clinic, 9500 Euclid Ave., Cleveland, OH 44195, USA; zimmere3@ccf.org (E.Z.); hastilk@ccf.org (K.H.)

**Keywords:** Parkinson’s disease, stair ambulation, gait biomechanics, postural instability and gait difficulties

## Abstract

**Background**: Stair ambulation is a complex motor task that presents a substantial fall risk for people with Parkinson’s disease (PwPD) who often have postural instability and gait difficulty (PIGD) and experience unpredictable freezing of gait (FOG) episodes. While dual-task (DT) interference during level walking is well-documented, its impact on stair ambulation, an everyday, high-risk activity, remains poorly understood. **Objective**: The aim of this study was to quantify the impact of dual tasking on patterns of motor control during stair ambulation using kinetic data from The Stair Ambulation and Functional Evaluation of Gait (Safe-Gait) system. **Methods**: Seventeen individuals with Parkinson’s disease (PD) completed three single-task (ST) and three dual-task (DT) trials on the Safe-Gait system, which sampled kinetic data via embedded force plates during stair ascent and descent. The force plate data were used to quantify step time, braking and propulsive impulses, and center of pressure (CoP) displacement and sway speed to assess DT effects on stair ambulation kinetics. **Results**: Dual-task conditions led to significant increases in step time (*p* < 0.001), braking impulse (*p* < 0.01), anteroposterior center of pressure (CoP) range (*p* < 0.05), and a decrease in mediolateral CoP speed (*p* < 0.01). **Conclusions**: Dual tasking during stair ambulation altered gait kinetics in PwPD, evidenced by slower, less stable movement patterns. These findings highlight the impact of cognitive motor DT interference on functional mobility and support the use of instrumented stair assessments to guide therapeutic care and fall risk interventions.

## 1. Introduction

Parkinson’s disease (PD) is a progressive neurodegenerative disorder characterized by four cardinal motor symptoms: tremor, bradykinesia, rigidity, and postural instability and gait difficulty (PIGD), all of which are exacerbated when a secondary cognitive task is introduced [1,2]. Postural instability and gait difficulties are particularly consequential as they substantially increase fall risk, often leading to fractures, hospitalizations, and declines in functional mobility and independence [2,3]. More than 60% of people with Parkinson’s disease (PwPD) experience falls annually [4,5], with most of these incidents occurring during everyday mobility tasks such as turning, navigating confined spaces, or ambulating stairs [6]. Stair ambulation poses substantial motor control challenges as it requires precise weight transfer, multi-joint coordination, and control of braking and propulsive forces to maintain postural stability during task execution [7]. For PwPD, these demands can exceed their neuromotor capacity, making stairs a particularly high-risk activity that can result in injurious falls [8].

The increased fall risk in PwPD is driven by the underlying neuropathological mechanisms of the disease. Parkinson’s disease is characterized by progressive degeneration of dopaminergic neurons in the basal ganglia and disruption of associated basal ganglia–thalamocortical circuitry [9]. This neural circuitry plays crucial roles in movement initiation, scaling, and automatic regulation [10]. With degeneration of dopaminergic neurons, control of fluid, precise movement becomes less automatic [10], increasing compensatory prefrontal and parietal cortex resources for motor execution [11]. The pathological reduction in automaticity disrupts gait timing, diminishes postural stability, and increases limb movement variability [12]. Additionally, the basal ganglia—particularly the striatum—plays an essential role in integrating sensorimotor information, including afferent proprioceptive inputs necessary for dynamic balance and safe stair ambulation [13].

Furthermore, PD-associated motor dysfunction is exacerbated under dual-task (DT) conditions. When PwPD simultaneously perform cognitive and motor tasks, there are significant deteriorations in gait speed, lower-extremity force generation, and postural control [2]. This DT interference reflects competing demands placed on already compromised neuromotor systems. Given PwPD’s reduced movement automaticity and increased reliance on cortical resources, the division of attention manifests in motor breakdown during DT performance [14]. Stair ambulation under DT conditions presents a particularly demanding scenario. Unlike level walking, stair ambulation requires precise foot placement, enhanced postural and braking control, and greater vertical force production, which all have minimal margins for error, especially when cognitive resources are divided [15]. Despite these challenges, stair ambulation remains relatively understudied in PD, particularly under DT conditions.

Current clinical mobility assessments predominantly occur on level surfaces in controlled environments and typically yield a single outcome measure [16]. While these evaluations can detect gross gait speed impairments, they fail to capture the nuances of real-world mobility challenges faced by PwPD. Moreover, standard clinical assessments rarely incorporate DT conditions or functional transitions like stair ambulation, scenarios that frequently trigger falls [17].

The gap in current assessment capability highlights the need for tools that offer quantitative biomechanical data from an ecological assessment. The Stair Ambulation and Functional Evaluation of Gait (Safe-Gait) system is a custom-built instrumented stair platform with integrated force plates that captures detailed biomechanical data on functional mobility. This system enables a direct quantitative evaluation of critical gait variables and can provide insight into specific postural control and functional mobility impairments. Importantly, Safe-Gait accommodates DT paradigms, allowing for an assessment of cognitive-motor interference during stair ambulation.

The aim of this project was to characterize the effects of dual tasking on the kinetics of stair ambulation in PwPD using the Safe-Gait system. This project addresses an important gap in understanding how PD impacts the control and coordination of functional locomotor activities such as stair ambulation under conditions that reflect those experienced in real-world environments. Data collected via the Safe-Gait system may broaden the understanding of PD mobility beyond straight-line walking and reflect real-word mobility demands, potentially facilitating informed treatment models to match the nuances of gait (dys)function.

## 2. Methods

### 2.1. Participants

Seventeen PwPD participated in this project (Table 1). Inclusion criteria included (1) a diagnosis of idiopathic PD, (2) proficient motor function to ambulate stairs, walk, and turn unassisted, and (3) proficient cognitive function to follow two-step commands. Exclusion criteria included (1) a diagnosis of dementia or any non-PD neurological condition or (2) a musculoskeletal condition affecting gait and balance. This study was approved by the Cleveland Clinic IRB, and all participants provided informed consent.

### 2.2. Safe-Gait System

**Figure 1:**
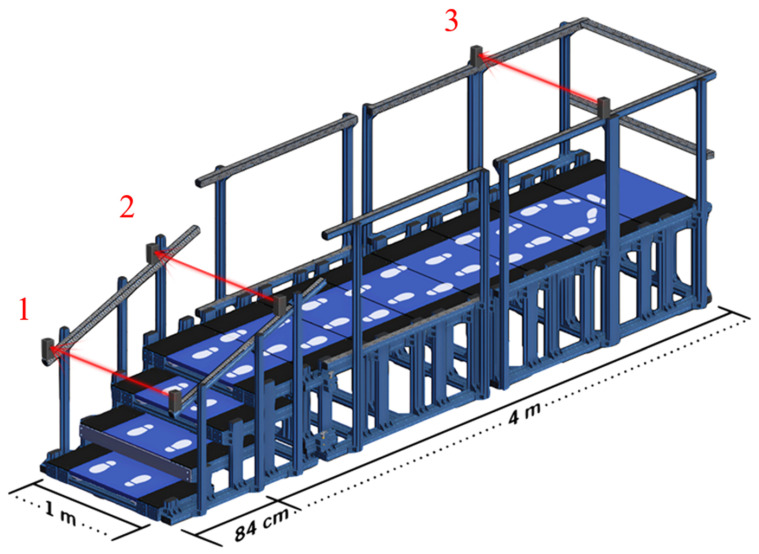
The Stair Ambulation and Functional Evaluation of Gait (Safe-Gait) platform consists of three standard steps and a 4 m flat surface, with reinforced handrails. It integrates force plates and photoelectric timing gates for comprehensive functional mobility assessment. Phases of the task are segmented by the three photoelectric timing gates (red beams): gates 1 and 2 for stair ascent and descent phases, gates 2 and 3 for walk-out and walk-back phases, and gate 3 for the turning phase.

The Safe-Gait system represents a functional mobility task that requires stair ambulation (ascending and descending) along with flat surface walking and a 180-degree turn. The perimeter of the Safe-Gait system has reinforced handrails to ensure participant safety during the task. The starting position and stairs of the Safe-Gait have embedded force plates. The force plates record at a sampling frequency of 1000 Hz using piezoelectric 3-component sensors (Kistler Group, Type 9260AA, Winterthur, Switzerland). Photoelectric laser timing gates are integrated into a custom-built staircase and walkway to provide temporal data for the five phases of the task. The photoelectric sensor gates, each comprising a photoelectric transmitter and a photoreceiver, are connected to a raspberry pi running a custom Python 3.13 data acquisition program on Raspberry Pi OS-Lite. Sensor states are monitored via a Measurement Computing USB-201 DAQ device (Digilent, Pullman, WA, USA), which timestamps when a participant enters and exits through each infrared beam, segmenting the assessment into five distinct phases. The ascent phase begins at gate 1 and ends at gate 2. The departure phase begins at gate 2 and ends at gate 3. The turn phase begins and ends at gate 3. The return phase begins at gate 3 and ends at gate 2. The descent phase begins at gate 2 and ends at gate 1 (Figure 1). A two-color stack light provides a visual cue to the participant to initiate the task. The force plate and timing gate data are synchronized via a control computer, using Vicon Nexus 2.16 software (Vicon Motor Systems, Oxfordshire, UK), that sends trial initiation and termination signals to both systems via a shared Wi-Fi network.

### 2.3. Experimental Protocol

Prior to data collection, the task requirements were explained to the participant and demonstrated by a member of the study team. The participant was allowed to use the handrails at their discretion to ensure safety, and a shared decision-making process was used to determine if the participant would complete the trials, informed by the participant’s level of comfort and the expertise of the clinician. Following the familiarization, the participant completed six trials on the Safe-Gait system, three single task (ST) and three DT. The participant began each trial when the light on the Safe-Gait system turned from red to green. For each trial, the participant ascended three stairs, walked to the end of the 4 m platform, completed a 180° turn, and returned to the starting position after flat-surface walking and stair descent. After completing the first ST trial, the participant was introduced to the DT condition. For each DT trial, the participant was given a random number between 100 and 200 and was instructed to verbally perform a serial 7s subtraction task, while simultaneously completing the previously described motor task. The administrator introduced the cognitive task and allowed the participant to practice the serial 7s subtraction until understanding and proficiency were demonstrated. The ST and DT trials were performed in an alternating sequence. All testing was completed in an off-medication state, operationally defined as at least 12 h after a participant’s last antiparkinsonian medication dose.

### 2.4. Kinetic Data

Kinetic data were obtained from force plates embedded in the stairs. Average step time was defined as the mean duration of the step-stance phase during stair ascent and descent. Braking impulse and propulsive impulse were defined as the area under the anteroposterior (AP) vertical ground reaction force (vGRF)-time curve during deceleration and push-off phases, respectively, normalized to body mass. Anteroposterior center of pressure (CoP) range was calculated as the average displacement in the AP direction per step during stair ascent and descent. Mediolateral (ML) CoP speed was defined as the average speed of CoP displacement in the ML direction during stair ambulation.

### 2.5. Statistical Analysis

The goal of the analysis was to determine if there was a difference between ST and DT conditions during a functional gait task and to characterize the kinetics of stair ambulation. Differences in tasks were assessed for the ascent and descent phases.

For each outcome, a separate linear mixed model (LMM) was constructed to assess differences in task, using fixed terms for task, phase, and the interaction between task and phase, and random intercepts by participant. For skewed outcomes, data were transformed to normality. Tests of significance were conducted using F-tests with Satterthwaite approximate denominator degrees of freedom. If the task by phase interactions were significant, pairwise contrasts were conducted to assess differences between tasks within each phase; otherwise, differences between tasks were assessed simultaneously across all phases. All statistical analysis was conducted using RStudio 2024.12.1, R version 4.4.3.

## 3. Results

There were no significant interactions between task condition and phase for any outcome (all *p* > 0.11), so differences in task were assessed for the collapsed phases (i.e., ascent and descent). During DT stair ambulation, participants exhibited notable kinetic differences compared to ST conditions. Under DT conditions, average step time increased by 16.4%, from 0.51 s to 0.60 s (*p* < 0.001). Normalized braking impulse increased by 13.9%, from 0.17 N·s/kg to 0.20 N·s/kg (*p* < 0. 001). Normalized propulsive impulse showed a smaller increase of 6.7%, from 0.20 N·s/kg to 0.22 N·s/kg, which did not reach statistical significance (*p* = 0.13). The AP center of pressure range increased by 8.3%, from 10.8 cm to 11.7 cm (*p* < 0.05). Lastly, ML average CoP speed decreased by 11.1%, from 1.07 m/s to 0.95 m/s (*p* < 0.001) (Table 2). The median time for ascent was 2.60 s [95% Confidence Interval (CI): 2.25, 2.99] under ST conditions and 3.20 s [2.78, 3.69] under DT conditions (23% greater under DT conditions, *p* < 0.0001). The median time for descent was 2.42 s [2.10, 2.79] under ST conditions and 2.85 [2.48, 3.29] under DT conditions (18% greater under DT conditions, *p* < 0.0001).

**Figure 2:**
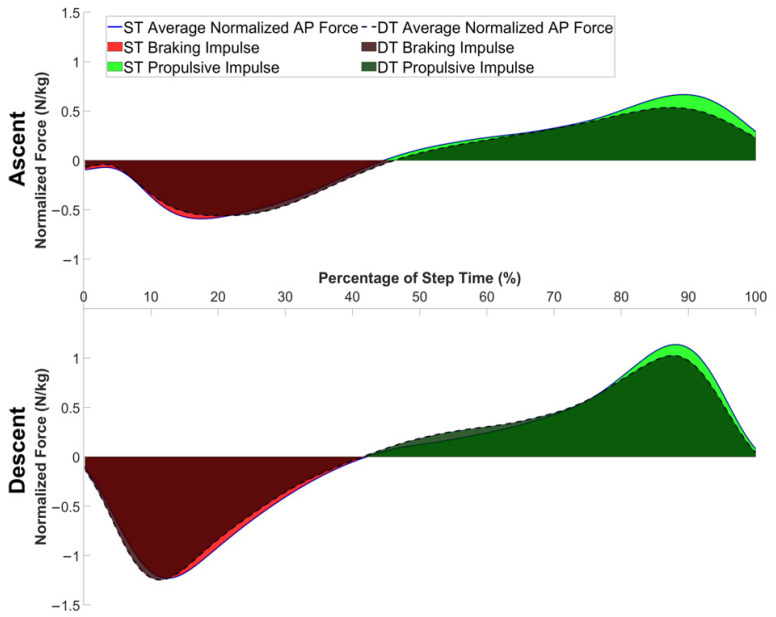
Anteroposterior ground reaction forces and impulse profiles during stair ambulation were impacted by dual-task conditions. Mean normalized AP force trajectories (N/kg) are shown for single-task (solid lines) and dual-task (dashed lines) conditions during stair ascent (top) and descent (bottom), plotted across step time (%). Shaded areas represent braking (red) and propulsive (green) impulses. Dual-task conditions resulted in greater braking impulse.

Figure 2 illustrates the anteroposterior ground reaction force profiles that underpin the data presented in Table 2. The force-time curves demonstrate the effects of DT conditions on force-generation patterns during stair ambulation. The expanded red shaded regions in the DT condition (dashed lines) represent the 17.6% increase in braking impulse quantified in Table 2. Similarly, the modest changes in the green shaded propulsive regions align with the 10% increase in propulsive impulse. The overall flattened and temporally extended force profiles under DT conditions reflect the significantly slower step times (17.6% increase) reported in the quantitative analysis.

## 4. Discussion

The results of this project demonstrate that dual tasking significantly alters stair ambulation kinetics in PwPD. Specifically, DT conditions led to longer step times, increased braking impulse, and decreased ML CoP speed. These adaptations reflect compensatory gait strategies in response to divided cognitive resources and impaired automaticity. The Safe-Gait system and force plate data were able to quantify these subtle but functionally relevant changes, underscoring its utility as a clinically feasible tool for detecting mobility impairments that standard overground assessments may miss, particularly under cognitively demanding conditions.

Participants exhibited longer step times, increased braking impulse, and slower ML CoP speed during DT stair ambulation. These findings align with previous research showing that DT conditions impair functional mobility in PwPD by taxing already compromised neural circuitry responsible for motor automaticity [18]. Notably, the observed increase in braking impulse without a corresponding reduction in propulsive force suggests a strategic reorganization of motor execution that emphasizes deceleration and postural control, a protective mechanism likely employed to prevent missteps and falls during cognitively demanding tasks [19]. This compensatory redistribution of force reflects a broader motor strategy of risk aversion when neuromotor resources are limited [20].

The slower step times observed under DT conditions are consistent with earlier reports showing temporal gait adjustments in PwPD as a compensatory adaptation when attention is diverted toward a secondary cognitive task [21]. This effect is especially pronounced in PwPD due to reduced basal ganglia function, which impairs automatic motor execution and necessitates additional recruitment of cortical resources [10]. The resulting competition for cortical processing capacity, which must now manage both cognitive and motor demands simultaneously, likely contributes to the slower, more cautious gait pattern reflected in the results here [22].

Changes in CoP kinetics further reinforce this interpretation. The reduction in ML CoP speed suggests constrained lateral movement, indicative of more cautious weight shifting strategies [23]. Concurrently, the increase in AP CoP range points to decreased control over forward momentum, a particularly critical impairment during stair descent when precise control is required to prevent forward falls [24].

These findings support the concept that DT conditions can reveal early or subtle gait abnormalities that may not be evident during conventional ST assessments. Current standard clinical assessments, such as Timed Up and Go (TUG), lack sensitivity to stair-specific or cognitive load-induced changes, limiting their utility in identifying fall risk in PwPD [11]. In contrast, the Safe-Gait system offers high resolution kinetic data under realistic functional conditions, enabling earlier detection of mobility impairments that may contribute to falls [25].

Although DT interference is well documented during level-ground, straight-line walking in PwPD, its amplification during more complex motor tasks such as stair ambulation suggests compounded risk for instability and falls in everyday environments [26,27]. Stair ambulation requires continuous sensorimotor integration, anticipatory postural adjustments, and spatial planning, all of which are susceptible to disruption in PwPD, particularly with increased cognitive load [28]. The Safe-Gait system evaluates gait kinetics during elevated-risk tasks (i.e., ambulating stairs while performing a concurrent cognitive task), without compromising participant safety. Thus, this approach provides a clinically feasible, ecologically valid framework for evaluating DT-associated mobility deficits in PwPD. The resultant data can be used to target rehabilitation strategies that may mitigate fall risk in individuals with PD. For example, greater change or impairment while ascending steps may indicate muscular deficiency for which targeted strengthening of the lower extremities should be a focus. Alternatively, difficulty descending the steps under DT conditions could trigger a rehabilitation intervention that is focused on improving dual-tasking capacity under complex conditions. From a rehabilitation perspective, the kinetic changes in gait observed in this study, such as prolonged braking phases and slowed ML CoP transitions, may offer important targets for therapeutic intervention. Programs emphasizing cognitive motor DT training, stair-specific exercises, and attentional cueing strategies can increase safety and improve everyday functional mobility in PwPD [29]. Additionally, the ability to quantify biomechanical responses to dual tasking provides a robust framework for individualized therapy planning and progress monitoring.

The high-resolution data provided by the Safe-Gait system presents an additional clinical utility: evaluating medication efficacy. While traditional dopaminergic therapies are often ineffective in managing PIGD symptoms, alternative pharmacological options, such as Schedule II stimulants like methylphenidate carry adverse risks [30,31]. Even modest improvements from safer dopaminergic agents may have meaningful clinical impact, particularly in reducing fall risk. The Safe-Gait system enables precise assessment of subtle medication-induced improvements in gait and postural stability, offering clinicians objective data to guide treatment decisions for patients with refractory PIGD symptoms.

In addition to its utility in assessing motor performance and guiding therapy in PwPD, the Safe-Gait system holds promise for broader application in other movement disorders and orthopedic populations. Abnormalities in gait kinetics are well documented in populations with multiple sclerosis and stroke, and these represent critical opportunities for targeted rehabilitation [32,33]. Access to high-resolution kinetic data may enable clinicians to develop more personalized and effective therapeutic strategies in these groups. Beyond treatment optimization, Safe-Gait may also support more accurate differential diagnosis—an enduring challenge in clinical neurology. Disorders such as Progressive Supranuclear Palsy–Parkinsonism Predominant (PSP-P) and Multiple System Atrophy (MSA) often mimic Parkinson’s disease in early stages, and subtle clinical differences can lead to prolonged misdiagnosis and inappropriate treatment. However, neuroimaging and gait analysis studies have demonstrated distinct patterns in both PSP-P and MSA when compared to PD [34,35,36]. Integrating objective kinetic data from systems like Safe-Gait may enhance diagnostic precision, thereby improving both clinical decision-making and patient outcomes.

The results of this study should be interpreted considering its limitations. The sample consisted predominantly of participants with relatively mild PIGD symptoms (majority of Hoehn & Yahr stage II), which may limit the generalizability of these findings to more severely impaired patients. Data was collected in the off-medication state on participants without an implanted deep brain stimulation (DBS) system. While the benefits of dopaminergic medications and DBS on tremor, rigidity, and bradykinesia symptoms are well understood, studies have reported inconsistent effects of these therapies in managing PIGD symptoms [31,37,38]. Future research should include participants with a broader range of symptom severities and therapy states to leverage high-resolution biomechanical data to explore the efficacy of these therapies in treating PIGD symptoms. Additionally, although ST trials served as an internal control condition, this study did not include a separate healthy older adult control group. Ongoing data collection in healthy older adults will help contextualize the observed impairments and clarify the extent to which they are PD-specific, and longitudinal studies are ongoing to explore biomechanical biomarkers that may be predictive of future falls.

The clinical utility of Safe-Gait lies primarily in its ability to quantify functional mobility in complex, real-world scenarios. By capturing detailed kinetic data during stair ambulation, this system bridges a critical gap between sophisticated research-grade biomechanical analysis and practical clinical assessment. Kinetic data collected with the Safe-Gait, such as increased braking impulse and slowed ML CoP speed, may serve as early indicators of reduced functional reserve in PwPD and may also improve the detection and treatment of non-PD movement disorders.

## 5. Conclusions

Stair ambulation poses a substantial risk for falls in older adults, and these risks are further amplified in individuals with neurological disorders such as PD. As such, the objective, high-resolution evaluation of postural and motor control during stair ambulation is critical for identifying patient-specific impairments and informing clinical decision-making. The Safe-Gait system offers a novel approach to functional mobility assessment by leveraging force plate instrumentation to quantify gait kinetics during stair ambulation—a task that better reflects the demands of real-world mobility than traditional level-ground walking assessments which are not representative of the multifaceted demands of everyday mobility.

Importantly, the Safe-Gait system enables the integration of dual-task paradigms into stair assessment, providing additional insight into cognitive motor interference and its contribution to functional impairment. This dual-task capability is particularly relevant in Parkinson’s disease, where divided attention frequently exacerbates gait instability and fall risk. Beyond the PD cohort described here, the system may also be valuable in the treatment and differential diagnosis of other neurological disorders, including multiple sclerosis, stroke, and atypical parkinsonian syndromes such as multiple system atrophy.

In summary, the Safe-Gait system represents a clinically meaningful advancement in the assessment of mobility necessary for community integration (e.g., stair ambulation and dual-task mobility). By capturing subtle yet consequential deficits in motor control, it offers the potential to improve diagnostic precision, enhance therapeutic targeting, and ultimately reduce fall risk across a range of neurological populations.

## Figures and Tables

**Table 1:** Participant demographics.

Demographics	Overall (N = 17)
**Age**	68.2 (7.8)
**Race**	
Black	3 (17.6%)
White	14 (82.4%)
**Sex**	
Female	5 (29.4%)
Male	12 (70.6%)
**Body Mass Index**	27.4 (4.4)
**Years of Education**	16.5 (1.8)
**Hoehn and Yahr stage**	
I	1 (5.9%)
II	10 (58.8%)
III	6 (35.3%)
**UPDRS Total Score**	41.7 (16.3)

Values reported as mean (SD) or number of participants (% of total participants).

**Table 2:** Compared to single-task conditions, dual-task conditions elicited significantly elevated step times, braking impulse, anteroposterior (AP) center of pressure (CoP) range and mediolateral (ML) CoP speed. Propulsive impulse also increased slightly under DT conditions, though this did not reach statistical significance.

Metric	ST	DT	Hedges’ *g*	*p*-Value
Average Step Time (s) ^A^	0.51, (0.44, 0.59)	0.60, (0.52, 0.69)	0.52	0.00015
Normalized Braking Impulse (N·s/kg)	0.17, (0.15, 0.20)	0.20, (0.17, 0.22)	0.48	0.0062
Normalized Propulsive Impulse (N·s/kg)	0.20, (0.18, 0.23)	0.22, (0.19, 0.24)	0.25	0.13
AP range (cm)	10.8, (9.4, 12.2)	11.7, (10.3, 13.1)	0.32	0.017
Average CoP Speed ML (m/s) ^A^	1.07, (0.90, 1.26)	0.95, (0.02, 1.12)	−0.35	0.0070

Data are presented as mean (95% CI) for approximately normally distributed data or median (95% CI) for skewed data. Hedges’ *g* is calculated on the transformed scale for skewed outcomes. ^A^ Skewed distribution.

## Data Availability

The raw data supporting the conclusions of this article will be made available by the authors upon request.

## Abbreviations

The following abbreviations are used in this manuscript:

PwPD	People with Parkinson’s disease
FOG	Freezing of gait
PIGD	Postural instability and gait difficulties
DT	Dual task
Safe-Gait	Stair Ambulation and Functional Evaluation of Gait
ST	Single task
CoP	Center of pressure
PD	Parkinson’s disease
AP	Anteroposterior
vGRF	Vertical ground reaction force
ML	Mediolateral
LMM	Linear mixed model
CI	Confidence interval
TUG	Timed Up and Go
